# Identification of Trogocytosis as an Essential Limitation Factor in hPSC-derived CAR Macrophages

**DOI:** 10.7150/ijbs.127434

**Published:** 2026-02-26

**Authors:** Baoqiang Kang, Shuoting Wang, Xinrui Guo, Huaisong Lin, Han Yan, Mingquan Wang, Tianhe Song, Zhishuai Zhang, Xing Hu, Yanling Zhu, Bo Feng, Jinfu Nie, Jiajun Liu, Guangjin Pan

**Affiliations:** 1 Centre for Regenerative Medicine and Health, Hong Kong Institute of Science and Innovation, Chinese Academy of Sciences, 15 Science Park West Avenue, Hong Kong Science Park, Hong Kong SAR, China.; 2 The Third Affiliated Hospital of Sun Yat-sen University, Guangzhou 510000, China.; 3 National Key Laboratory of Immune Response and Immunotherapy, Guangzhou Institutes of Biomedicine and Health, Chinese Academy of Sciences, Guangzhou, 510530, China.; 4 University of Chinese Academy of Sciences, Beijing 100049, China.; 5 Guangdong Provincial Key Laboratory of Stem Cell and Regenerative Medicine, Guangdong-Hong Kong Joint Laboratory for Stem Cell and Regenerative Medicine, Center for Cell Lineage and Cell Therapy, Guangzhou Institutes of Biomedicine and Health, Chinese Academy of Sciences, 510530, China.; 6 GIBH-HKU Guangdong-Hong Kong Stem Cell and Regenerative Medicine Research Centre, GIBH-CUHK Joint Research Laboratory on Stem Cell and Regenerative Medicine, Guangzhou Institutes of Biomedicine and Health, 510530, Chinese Academy of Sciences.; 7 School of Biomedical Sciences, Faculty of Medicine, CUHK-GIBH CAS Joint Research Laboratory on Stem Cell and Regenerative Medicine, The Chinese University of Hong Kong, Room 105A, Lo Kwee-Seong Integrated Biomedical Sciences Building, Area 39, Shatin, NT, Hong Kong SAR, China.; 8 Hefei Cancer Hospital of CAS, Institute of Health and Medical Technology, Hefei Institutes of Physical Science, Chinese Academy of Sciences (CAS), Hefei, China.

**Keywords:** Human pluripotent stem cells, CD5 CAR-Macrophage, Trogocytosis, T-cell malignancies, Immuno-therapy

## Abstract

Generation of CAR macrophages from induced pluripotent stem cells(iPSCs) hold great potential for immunotherapy, particularly against T-cell malignancies which are challenging in CAR-T therapy. However, the tumoricidal activity of human iPSCs derived CAR-macrophages (iCAR-Ms) remains less extensively analyzed. Here, we generated human iCAR-Ms targeting CD5 for T-cell malignancy therapy. iCAR-Ms show up-regulation of immunity related functions as well as tumoricidal activity against different T malignant cells expressing CD5. However, the tumoricidal activity of iCAR-Ms is highly related to CD5 density on tumor cells and depends on high dose treatment *in vivo*. We further reveal that the tumor cells resisting iCAR-M killing show reversible CD5 loss mediated by iCAR-M trogocytosis. In contrast, the retrieved iCAR-Ms from tumor cell co-culture retained tumoricidal activity on new tumor cell expressing CD5. Thus, we identify trogocytosis as an important limiting factor on iCAR-Ms therapy, providing a rationale for developing enhanced CAR-M therapies.

## Introduction

T cell-based immunotherapy, exemplified by chimeric antigen receptor (CAR)-T therapy, represents one of the most promising approaches for cancer treatment 1, 2. CAR-T therapy has demonstrated significant clinical efficacy against B-cell malignancies, leading to the approval of several CD19- and B-cell maturation antigen (BCMA)-targeted CAR-T products 3, 4. In contrast, T-cell malignancies comprise a group of disorders characterized by dysfunctional clonal expansion at various stages of T-cell development 5. Conventional treatments for T-cell malignancies remain highly challenging, with five-year survival rates around 30-50% 6, 7. Applying CAR-T therapy to target T cell marker such as CD5, CD7 to treat T-cell malignancies has been attempted, but is challenging due to the scarcity of autologous normal T cells and the risk of CAR-T cell fratricide 8-11. Besides, solid tumors formed by T-cell lymphomas, along with their tumor microenvironment, pose significant barriers to CAR-T cells infiltration and exhibit immunosuppressive properties.

Beyond T-cell therapy, other immune effectors such as N K cells and macrophages have attracted increasing attention for immunotherapy, especially for malignancies resistant to T cell-based approaches 12. Macrophages play essential roles in innate immunity, participating in processes such as apoptotic cell clearance, homeostasis maintenance, and immune cell activation 13, 14. In addition, macrophages have a better infiltration and tumor tissue residence than T cells and NK cells. Engineering macrophages to express chimeric antigen receptors (CAR-Ms) has emerged as a promising strategy for treating various tumors, particularly solid tumors due to their better tumor tissue residence 15-17. Human CAR-Ms have been generated using THP-1 monocytic cells or peripheral blood mononuclear cells (PBMC)-derived macrophages, and exhibit enhanced phagocytic capability against target tumor cells 18, 19. CAR-Ms targeting antigens such as CD19 and HER2 have shown promising antitumor effects in mouse models 16, 17, 20, 21. Given the limited availability of peripheral blood or cord blood-derived macrophages, human pluripotent stem cells (PSCs) offer an unlimited source for macrophage-based therapies. PSC-derived macrophages expressing CARs targeting CD19, HER2, PSCA, EGFRvIII, and Glypican-3 have been reported to suppress tumor growth in mouse models 22-25. However, most studies require a high effector-to-target (E: T) ratio for significant tumor suppression 16, 17, 19, 24, 25, suggesting unknown mechanisms limiting CAR-M tumoricidal activity.

Trogocytosis is a dynamic process by which immune cells actively nibble and acquire membrane fragments from target cells through the formation of an immunological synapse 26. This phenomenon leads to functional alterations in both recipient and donor cells, thereby mediating a variety of physiological effects 27, 28. In CAR-T therapy for hematological malignancies, trogocytosis has been implicated as one of the key mechanisms driving antigen-negative relapse and treatment resistance 29-31. While CAR-M possess potent phagocytic capacity, it is critical to evaluate and regulate their trogocytic activity to balance effective tumor killing against detrimental trogocytosis, which may otherwise sustain antitumor efficacy and limit tumor escape. However, trogocytosis in CAR-M killing tumor cells remains less studied.

In this study, to investigate the potential of macrophage therapy against T-cell malignancies, we generated human iPSC-derived CAR-Ms (iCAR-Ms) targeting CD5, a universal marker for T cells for the treatment of T-cell malignancies. Our results demonstrate that the tumoricidal activity of iCAR-Ms depends heavily on a high E:T ratio and CD5 density on tumor cells. The tumor cells that resist iCAR-M killing show CD5 loss mediated by iCAR-M trogocytosis. Thus, trogocytosis represents a major factor limiting the tumoricidal efficacy of iCAR-Ms and facilitating tumor escape.

## Results

### Generation of human CAR-Ms targeting CD5 from iPSCs

**Figure 1:**
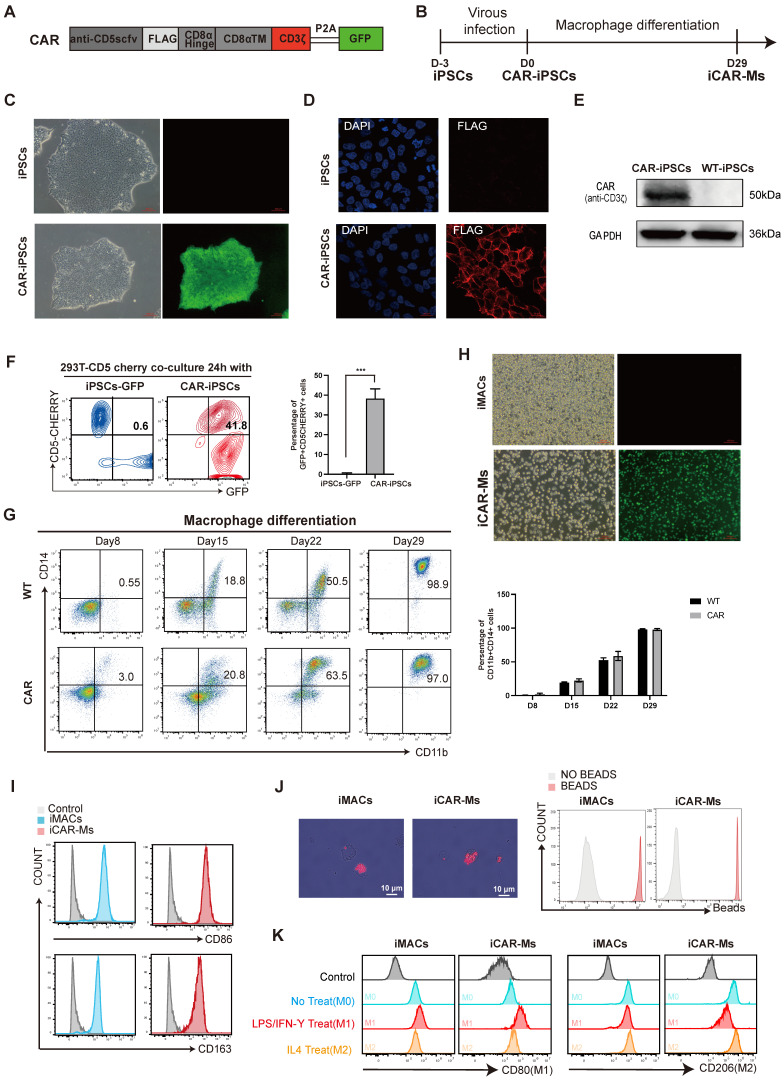
** Generation of human iCAR-Ms targeting CD5. (A)** Construction of CD5-targeting CAR macrophages. **(B)** Schematic strategy for generating anti-CD5 CAR-Macrophage from human iPSCs. **(C)** The morphology and green fluorescent protein expression on indicated cells, Scale bar: 100μm. **(D)** Immunofluorescence staining analysis of CAR expression in iCAR-Ms. Blue, DAPI; Red, flag. Scale bar: 20μm. **(E)** Immunoblot analysis of indicated protein expression levels in CAR-iPSCs and WT-iPSCs. **(F)** FACS analysis of the GFP^+^ CAR-iPSCs and CD5cherry^+^ 293T-CD5cherry in coculture for 24h. The data represent mean ± SD from three independent biologic replicates (n = 3). **(G)** FACS analysis of the indicated markers during macrophage differentiation. The data represent mean ± SD from three independent biologic replicates (n = 3). **(H)** The morphology and green fluorescent protein expression of indicated cells, Scale bar: 100μm.** (I)** FACS analysis the indicated macrophage markers in mature iMACs and iCAR-Ms. Undifferentiated human iPSCs serve as control. **(J)** Engulf of red fluorescent latex labeled beads by iMACs and iCAR-Ms phagocytosis examined by confocal microscopy (left) and FACS. Scale bar: 10μm. **(K)** FACS analysis of M1 or M2 surface markers in polarized iMACs and iCAR-Ms by the indicated treatment. M1 polarization was induced by LPS/ IFN-Ƴ while M2 polarization by IL4.

To develop an immunotherapy for T-cell malignancies, we constructed a lentiviral vector encoding a CD3ζ-containing CAR targeting CD5 (**Fig.1A-B**). We also made a CAR construct with the deletion of CD3ζ, the critical intra-cellular domain in typical CAR construct as a control (**Fig.S1A-B**). The CD5_CAR and mutant CAR(CAR∆) were introduced into human iPSCs that was previously generated in our laboratory from CD34⁺ cord blood hematopoietic stem and progenitor cells 32-36 (**Fig.1C, Fig.S1C-D**). Immunostaining for the inserted FLAG tag or GFP, together with western blot analysis, confirmed homogeneous CAR expression on the cell surface and the expected size (**Fig. 1C-E**). To assess whether the CAR specifically binds CD5, we co-cultured CAR-iPSCs or CAR∆ iPSCs with 293T cells overexpressing CD5-mCherry. FACS analysis revealed that either CAR-iPSCs or CAR∆ iPSCs substantially bound 293T-CD5-mCherry cells (**Fig. 1F, Fig.S1E**). We then generated macrophages from CAR-iPSCs or CAR∆ iPSCs based on our previously published protocol 32-36. Macrophages induced from CAR-iPSC (iCAR-Ms) or CAR∆ iPSCs (iCAR∆-Ms) expressed typical macrophage markers such as CD14, CD11b, CD80, CD86, CD163, CD206 and showed substantial bead-engulfment capability (**Fig. 1G-J, Fig.S1F-J**). iCAR-Ms could also be polarized toward M1 or M2 phenotypes upon LPS or IL-4 treatment, demonstrated by differential typical markers CD80 for M1 or CD206 for M2 (**Fig. 1K**). In short, iCAR-Ms that specifically bind CD5 were generated from human iPSCs.

### iCAR-Ms show up-regulation of pro-immunity genes

**Figure 2:**
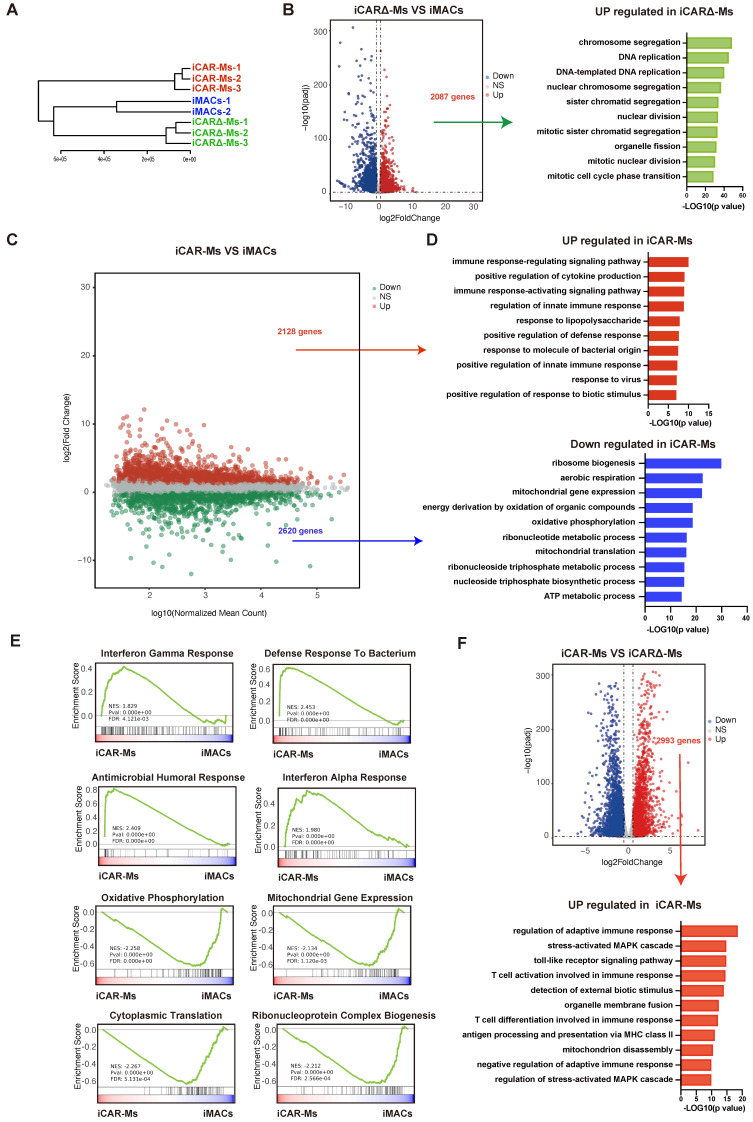
** iCAR-Ms show up-regulation of pro-immunity genes. (A)** Hierarchical clustering dendrogram of RNA-seq samples. **(B)** Volcano plot for genes expression in iCAR∆-Ms and iMACs. The y-axis shows -log10 Padj; x-axis shows log2 transformation of fold change and red points indicate significant up-regulated genes(left). Gene Ontology (GO) terms enriched in genes upregulated in iCAR∆-Ms. **(C)** MA plot for genes expression in iCAR-Ms and iMACs. The x-axis shows log10 mean; y-axis shows log2 transformation of fold change and red or green points indicate significant genes. **(D)**Top Gene Ontology (GO) terms enriched in genes up-regulated(left) and down-regulated in iCAR-Ms. **(E)** GSEA analysis for iCAR-Ms and iMACs. **(F)** Volcano plot for genes expression in iCAR-Ms and iCAR∆-Ms. The y-axis shows -log10 Padj; x-axis shows log2 transformation of fold change and red points indicate significant up-regulated genes(top). Gene Ontology (GO) terms enriched in genes upregulated in iCAR-Ms(bottom).

To extensively analyze iCAR-M, we performed transcriptomes in iCAR-Ms as well as wild type iMac and iCAR∆-Ms for comparison (**Fig.2A**). Compared with WT iMACs, the up-regulated genes in iCAR∆-Ms showed no enrichment on immunity related genes (**Fig.2B**). In contrast, the up-regulated genes in iCAR-Ms were enriched in immuno-defense and pro-immunity related genes (**Fig.2C-D**). While, ribosome biogenesis as well as energy metabolism genes were down-regulated in iCAR-Ms (**Fig.2C-D**). We then performed side-by-side comparative analysis between iCAR-Ms and iCAR∆-Ms. Based on Gene Set Enrichment Analysis (GSEA) analysis, the gene set with the function of interferon gamma response, interferon alpha response and defense response to bacterium as well as regulation of adaptive immunity were highly enriched iCAR-Ms while not iCAR∆-Ms (**Figure 2E-F**). Further RT-qPCR analysis confirmed that the expression of genes involved in inflammation was upregulated in iCAR-Ms (**Fig.S1K**). These data demonstrate that CAR encoding CD3ζ up-regulates genes with immunity regulatory function in macrophages.

### iCAR-Ms exhibit CD5-dependent tumoricidal activity

**Figure 3:**
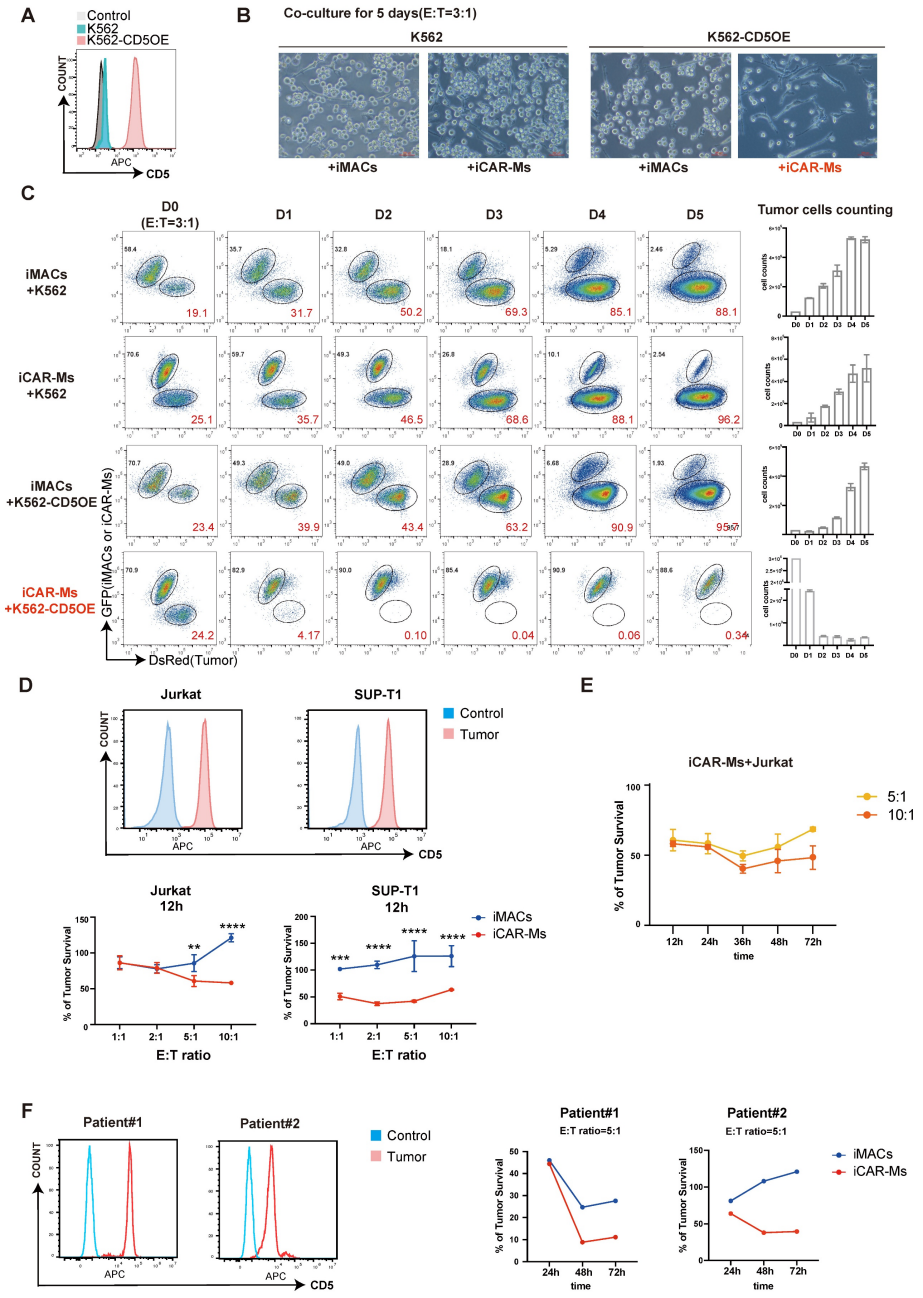
** iCAR-Ms exhibit CD5-dependent tumoricidal activities. (A)** FACS analysis of CD5 expression on K562 cells with CD5 overexpression, unstained K562 served as control.** (B)** Co-culture of WT K562 or K562-CD5OE cells with iMACs or iCAR-Ms. The pictures were taken on indicated cells at 5 days of co-culture, Scale bar: 200μm. **(C)** FACS analysis of DsRed^+^ WT K562 or K562-CD5OE cells with iMACs or iCAR-Ms. The remaining tumor cells were counted at indicated time point during co-culture. These data represent mean ± SD from three independent replicates (n=3). **(D)** Killing assay on indicated T-cell tumor lines. FACS analysis of CD5 expression on indicated cells(top). Killing assay of indicated tumor by iCAR-Ms or iMACs after 12h of co-culture at indicated E:T ratios (bottom). These quantitative data represent mean ± SD from three independent replicates (n=3). **(E)** Killing assay of Jurkat by iCAR-Ms at E: T=5:1 or 10:1 after indicated time of co-culture. These data represent mean ± SD from three independent replicates (n=3). **(F)** FACS analysis of CD5 expression on peripheral blood mononuclear cells from T-ALL patients, unstained cells served as control(left). Killing assay of indicated cells by iCAR-Ms or iMACs as control at E: T=5:1 after indicated time of co-culture.

To evaluate the tumor-suppressive function of CD5-targeting iCAR-Ms, we first overexpressed CD5 in K562 cells, which do not express CD5 endogenously (**Fig. 3A**). iCAR-Ms significantly suppressed the growth of CD5-expressing K562 cells (K562-CD5), while not wild-type 37 K562 cells (**Fig. 3B-C**). In contrast, WT iMACs and iCAR∆-Ms showed little suppression on either of these two cell types (**Fig. 3B-C, Fig.S2A-B**), indicating that iCAR-Ms specifically target cells with CD5-expression. We next examined the suppressive effects of iCAR-Ms on T-cell tumor lines endogenously expressing CD5, such as Jurkat and SUP-T1 cells (**Fig. 3D**). iCAR-Ms effectively suppressed survival of Jurkat and SUP-T1 cells after 12 hours of co-culture at various E:T ratios (**Fig. 3D, Fig. S2C-D**). However, a substantial proportion of tumor cells resisted iCAR-M-mediated killing even at the highest E:T ratio tested (**Fig. 3D**). Prolonging co-culture time did not further enhance suppression on tumor survival at higher E:T ratios (**Fig. 3E**). We also isolated primary tumor cells from two T-cell acute lymphoblastic leukemia (T-ALL) patients and tested their sensitivity to iCAR-Ms. iCAR-Ms suppressed primary tumor cells from both patients, with more suppression on tumor cells from Patient 1, which expressed higher CD5 levels (**Fig. 3F**). Together, these results demonstrate that iCAR-Ms targeting CD5 display CD5-dependent but limited tumoricidal activity against T malignant cells.

### iCAR-Ms show dose dependent tumor-suppressive effects *in vivo*

**Figure 4:**
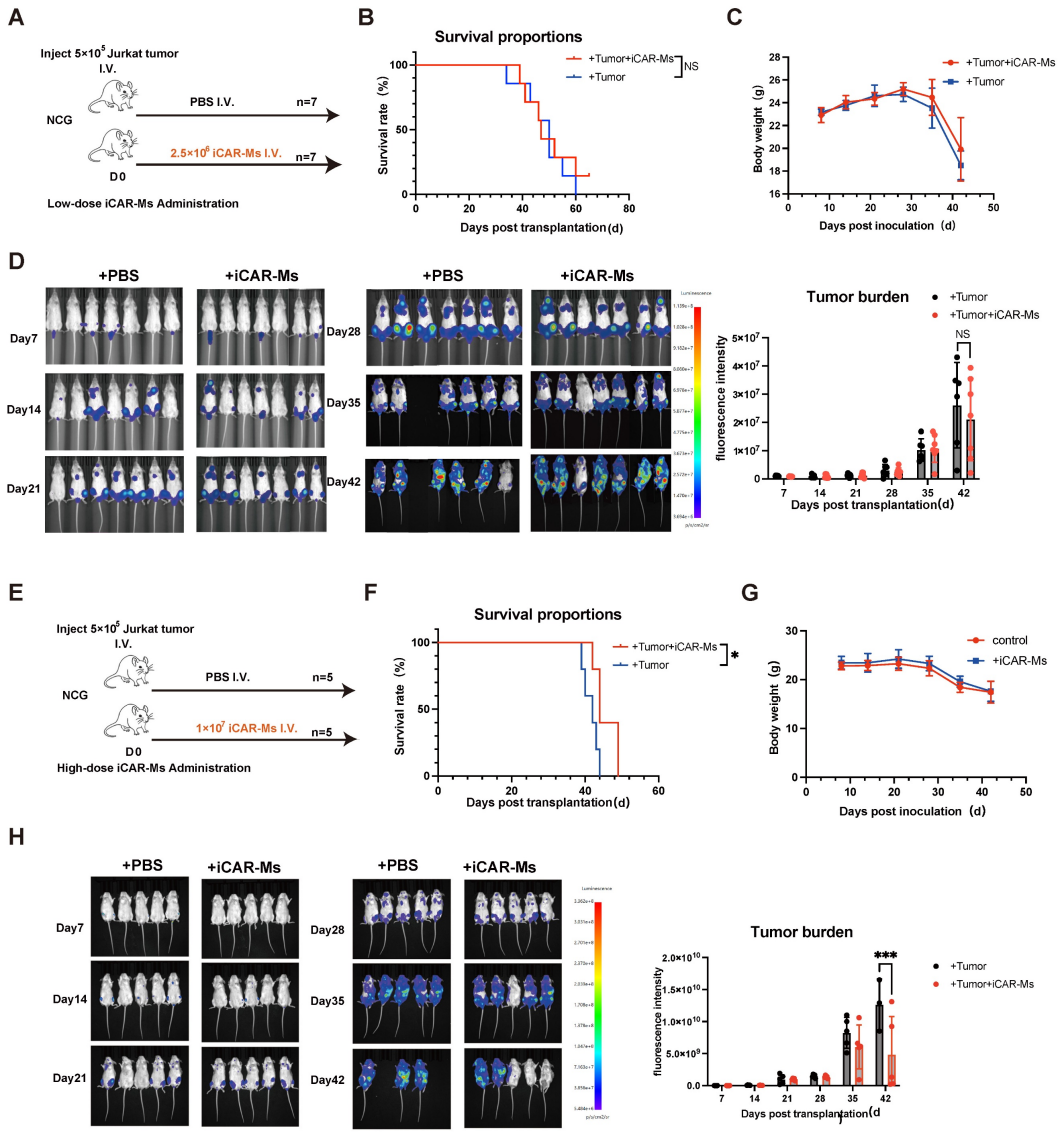
** iCAR-Ms show tumor-suppressive effects in *vivo* at higher dose treatment. (A)** Experimental design: NCG mice were IV. injected with luciferase_Jurkat and treated with IV PBS or iCAR-Ms as shown. **(B)** Kaplan-Meier survival curve for animals injected with Jurkat tumor cells and indicated treatments. Statistical significance was calculated using the log-rank Mantel-Cox test.** (C)** Mouse weight monitoring. The weight of mice (fold) was calculated by measuring weight/initial weight. Data are represented as mean ± SD. **(D)** Imaging of luciferase-expressing Jurkat tumor-bearing mice (left). Tumor burden was measured by bioluminescence (total flux) at indicated time. Data are represented as mean ± SD. **(E)** Experimental design: NCG mice were IV. injected with luciferase_Jurkat and treated with IV PBS or iCAR-Ms as shown. **(F)** Kaplan-Meier survival curve for animals injected with Jurkat tumor cells and indicated treatments. Statistical significance was calculated using the log-rank Mantel-Cox test.** (G)** Mouse weight monitoring. The weight of mice (fold) was calculated by measuring weight/initial weight. Data are represented as mean ± SD. **(H)** Imaging of luciferase-expressing Jurkat tumor-bearing mice (left). Tumor burden was measured by bioluminescence (total flux) at indicated time. Data are represented as mean ± SD.

We then examined the tumor-suppressive effect of iCAR-Ms in a xenograft mouse model of T-ALL. NSG mice were intravenously injected with 5 × 10⁵ luciferase-expressing Jurkat T-cell malignancies cells, followed by injection of 2.5 × 10⁶ or 1x10^7^ iCAR-Ms one day later (**Fig. 4A, E**), based on other reported studies 17, 19-21, 24, 25. To evaluate the tumor killing of iCAR-Ms *in vivo*, we used a single injection of iCAR-Ms. Compared to PBS-treated controls, low dose iCAR-M treatment did not significantly promote overall survival of animals (**Fig. 4B**), whereas higher dose treatment did show measurable survival benefits (**Fig. 4F**). iCAR-Ms treatment also showed suppression on tumor growth as detected by monitoring bioluminescence imaging (BLI) in live animals compared with PBS-treated groups, (**Fig.4D, H**). However, no statistically significant difference in tumor burden was observed between iCAR-Ms and PBS-treated group in low dose animal trial (**Fig. 4D**). Notably, there were no significant body weight loss in mice injected with iCAR-M relative to PBS control in low or high dose group **(Fig.4C, G)**, indicating the low systemic toxicity for iCAR-M treatment. We also examined the function of iCAR-Ms in another T-cell lymphoma, SUP-T1 based on the xenograft model. NSG mice receiving SUP-T1 cells (5 × 10⁵, luciferase-expressing) followed by iCAR-Ms (1 × 10⁷, 2 days later) showed significant suppression of tumor growth and burden compared to PBS controls** (Fig. S4A-C)**. This efficacy was accompanied by a survival benefit and no significant body weight loss** (Fig. S4D-E)**. Together, these data suggest that iCAR-Ms exert tumor-suppressive effects in *vivo*, but are largely dependent on the cell doses.

### Trogocytosis contributes to tumor escape from iCAR-M killing

**Figure 5:**
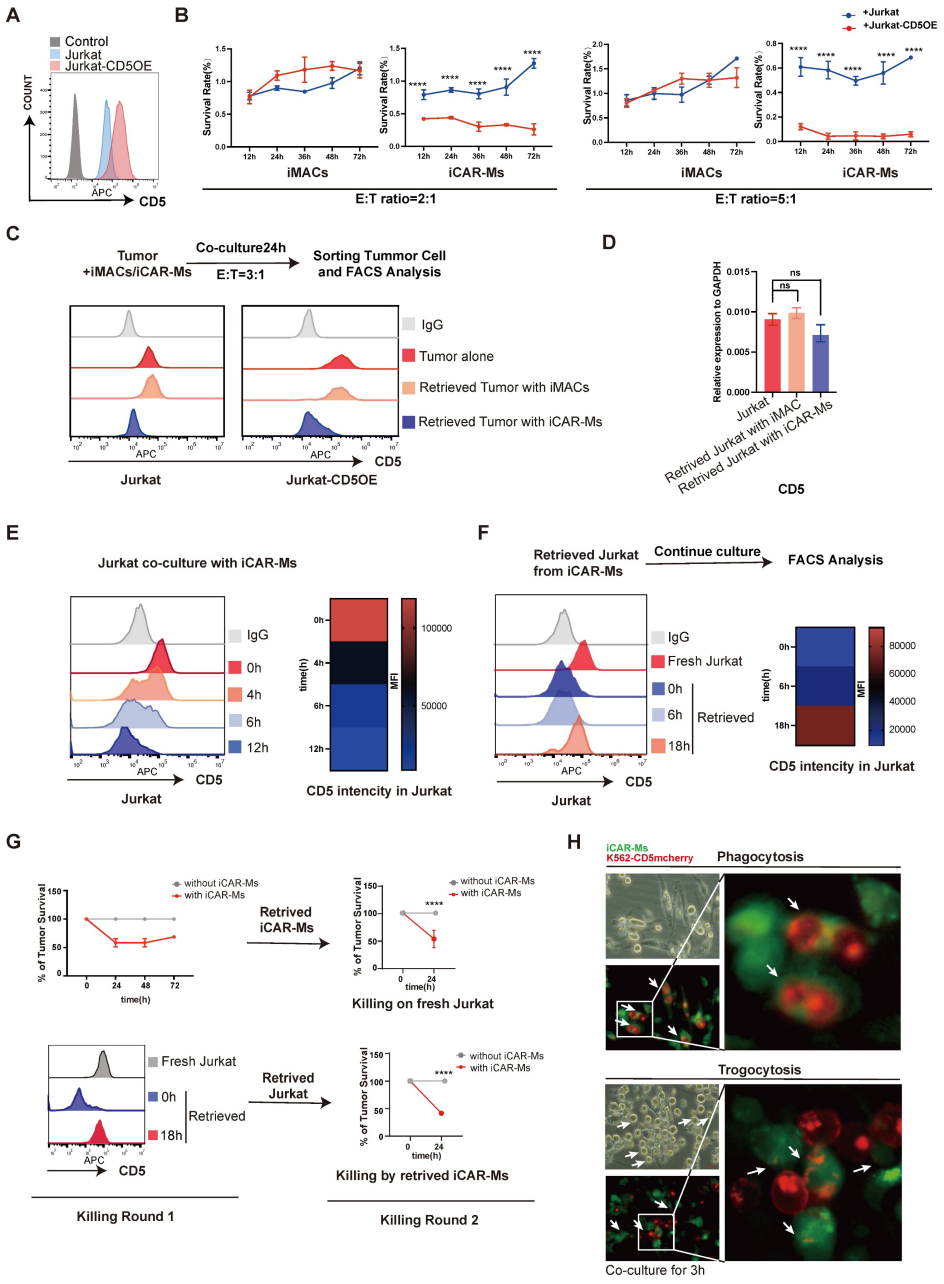
** Trogocytosis contributes to tumor escape from iCAR-M killing. (A)** FACS analysis of CD5 expression on Jurkat cells with CD5 overexpression, unstained Jurkat served as control. **(B)** Killing assay of indicated cells by iCAR-Ms or iMACs at E: T=5:1 or 2:1 after indicated time of co-culture. These data represent mean ± SD from three independent replicates (n=3).** (C)** Analysis of CD5 expression on retrieved tumors from iMAC/iCAR-Ms co-culture. Tumor cells were retrieved by FACS sorting from co-culture with iMACs or iCAR-Ms (top). FACS analysis of CD5 expression on indicated cells(bottom). **(D)** RT-qPCR analysis of the CD5 expression in indicated cells. These data represent mean ± SD from three independent replicates (n=3). **(E)** Jurkat cells rapidly loss CD5 upon culture with iCAR-Ms. FACS analysis and heatmap of CD5 density on Jurkat co-cultured with iCAR-Ms at indicated time. **(F)** Retrieved Jurkat cells regain CD5 upon further culture. Flow chart representing the culture of retrieved Jurkat(top). FACS analysis and heatmap of CD5 density on indicated cells(bottom). **(G)** 2 rounds killing examinations on retrieved Jurkat cells or iCAR-Ms. Left, killing assay 1: Killing assay of Jurkat by iCAR-Ms at E: T=5:1(top) and FACS analysis of CD5 expression on indicated cells(bottom); Right, killing assay 2: Killing assay of fresh Jurkat by retrieved iCAR-Ms at E:T ratio 5:1(top). Killing assay of retrieved 18h Jurkat by iCAR-Ms at E:T ratio 5:1(bottom). **(H)** Fluorescence microscopy images of phagocytosis(top) and trogocytosis(bottom) after iCAR-Ms and K562-CD5mcherry co-culture 3h. Green: iCAR-Ms, Red: K562-CD5mcherry. Scale bar: 20μm.

To investigate mechanisms limiting iCAR-M tumoricidal activity, we overexpressed CD5 in Jurkat cells to elevate CD5 levels (**Fig. 5A**). iCAR-Ms showed enhanced suppression of CD5-overexpressing Jurkat cells compared to WT Jurkat cells at both low and high E:T ratios (**Fig. 5B**). WT iMACs did not suppress the survival of either cell type (**Fig. 5B**). The escaped tumor cells from iCAR-M killing were retrieved and analyzed by FACS (**Fig. 5C**). Both WT and CD5-overexpressing Jurkat cells that escaped iCAR-M killing showed substantial reduction in CD5 levels, while not upon co-culture with WT iMACs (**Fig. 5C**). Notably, the survived CD5-overexpressing Jurkat cells from iCAR-M killing also showed more CD5 levels compared with WT Jurkat cells, which may explain that they were more sensitive to iCAR-M killing (**Fig. 5C**).

Interestingly, CD5 mRNA levels did not decrease significantly in escaped Jurkat cells from iCAR-M killing (**Fig. 5D**). Time-course analysis revealed rapid reduction of CD5 protein intensity in Jurkat cells within 12 hours of co-culture with iCAR-Ms (**Fig. 5E**). Indeed, in a cycloheximide (CHX) chase assay, we detected a substantial decrease in CD5 protein half-life when co-culture with iCAR-Ms (**Fig. S3F**). Interestingly, the retrieved Jurkat cells from iCAR-M co-culture resisting the killing re-gain CD5 protein upon further culture alone (**Fig. 5F**). These re-cultured Jurkat cells with CD5 re-expression were susceptible to the killing by iCAR-Ms retrieved from the first co-culture (**Fig. 5G**). In addition, the retrieved iCAR-Ms from the first round of tumor killing culture also maintained tumoricidal activity against fresh Jurkat cells (**Fig. 5G**). These data demonstrate that tumor escape from iCAR-M killing is primarily due to reversible loss of CD5 in tumor cells rather than iCAR-M exhaustion. Trogocytosis has been reported in CAR-T and CAR-NK cells, leading to antigen loss and killing escape 30, 31. We therefore investigated whether iCAR-Ms mediate trogocytosis resulting in the reversible CD5 loss. We constructed a CD5-mCherry fusion protein and overexpressed it in K562 cells via lentiviral transduction** (Fig. S3A-C)**. CD5 signal was observed in iCAR-Ms but not in iMACs by flow cytometric analysis after co-culture. **(Fig.S3D)**. Time-lapse imaging of co-cultures between GFP-labeled iCAR-Ms and K562-CD5-mCherry cells revealed both phagocytosis (whole-cell engulfment) and trogocytosis (transfer of CD5-mCherry followed by tumor cell release) (**Fig. 5H and Movies S1-3**). Together, these data demonstrate the antigen loss mediated by trogocytosis is an important limitation factor on tumoricidal activity of CAR-macrophages.

## Discussion

CAR macrophages have drawn significant research interests as a novel immunotherapy platform for cancer treatment 38, 39. CAR-Ms targeting various tumor antigens have shown promise effects based on *in vitro* as well as mouse models. Notably, CAR macrophages targeting HER2 and mesothelin have received FDA approval for clinical trials 40. Both PBMC-derived and PSC-derived macrophages have been used as sources for CAR-Ms that were demonstrated to hold antitumor effects 17, 19, 23-25. However, CAR-Ms often require high E:T ratios and additional strategies to achieve efficient tumor suppression* in vivo*, indicating limitations in their tumoricidal activity 15, 18, 20-22. However, the mechanisms underlying these limitations remain poorly understood.

In this study, we generated human iPSC-derived CAR macrophages targeting CD5 (iCAR-Ms) for treating T-cell malignancies. iCAR-Ms displayed limited tumoricidal activity that depended heavily on CD5 antigen density. Escaped tumor cells underwent reversible CD5 protein loss. We further demonstrated that iCAR-Ms mediate trogocytosis upon contact with tumor cells, leading to CD5 loss and tumor escape. Our findings identify trogocytosis as a major factor limiting the efficacy of iCAR-Ms and promoting tumor escape.

Trogocytosis involves the transfer of surface molecules between cells upon contact. In immune cells, trogocytosis regulates immune efficiency and adaptability, such as in antigen presentation 28. However, trogocytosis also occurs in CAR-T and CAR-NK cells, reducing tumor antigen density and promoting fratricide among CAR-expressing cells 27, 30, 31, 41. Macrophages are professional phagocytes that engulf and digest pathogens, cancer cells, and cell debris. In our study, *in vitro* experiments indicate that trogocytosis occurs when CAR-M engage with target tumor cells, leading to antigen loss on the target cell surface and consequent immune escape. Besides, the dose-dependency of CAR-M is not simply a linear matter of killing efficiency, but rather a kinetic competition between two processes: the killing by CAR-M; and escaping of tumor cells via trogocytosis. A low dose shifts the balance toward escape, leading to therapeutic failure, whereas a high dose shifts it toward rapid clearance, thereby preventing escape. This underscores precisely that trogocytosis constitutes a critical bottleneck limiting CAR-M efficacy, especially when effector cells are insufficient. CD3ζ, the intracellular domain derived from TCR signaling, is commonly used in CAR-M constructs and has been associated with improved antitumor effects 16, 19, 25, 42. In our study, CD3ζ-based CAR signaling induced trogocytosis and phagocytosis in iMACs. Deleting CD3ζ abolished both trogocytosis and phagocytosis, as well as tumor suppression (**Fig. S2C-D, Fig. S3D-E**). Transcriptomic profiling indicated that CD3ζ signaling upregulates immune regulatory genes, potentially enhancing antigen presentation via trogocytosis.

Strategies to modulate trogocytosis have been explored in CAR-NK and CAR-T cells, including actin remodeling inhibition and dual-targeting designs 43, 44. Our findings indicate that CAR-M therapy may be particularly unsuitable for single-target scenarios that are highly susceptible to antigen loss. Clinical strategies should prioritize targets with stable expression or employ dual/multi-target CAR-M constructs to counteract immune escape driven by trogocytosis-mediated antigen downmodulation. In the case of T cell malignancies, future exploration on combinations of different markers such as CD7 and CD5 might enable more sustained disease control. Notably, the observed threshold on the antitumor effect in animal model might not be available in clinical applications. Future studies need to identify critical factor to enhance the overall anti-tumor potency in CAR-Ms. Our findings highlight a rational for next-generation CAR-M design, overcoming incorporate trogocytosis during tumor cell killing. Unlike the standardized CAR-T design, the optimal intracellular signaling in CAR-M remains less studied. Various structures have been tested in CAR-M design, such as CD3ζ, FcRγ, as well as other innate immune receptors. Identifying macrophage-optimized intracellular signaling domains that preferentially induce phagocytosis rather than trogocytosis hold great potential in future studies to improve the tumoricidal efficacy of CAR-Ms.

## Methods

### Cell cultures

hiPSCs were maintained on Matrigel (1:100 dilution; BD)-coated plates in mTeSR1 medium (Stem Cell Technologies). Medium was changed every day and cells were passaged onto fresh Matrigel-coated plates every 3 days using 0.5 mM EDTA. hiPSCs were cultured under 37 °C, 20% O_2_, and 5% CO_2_ and had been tested to be free of mycoplasma contamination. In particular, the generation of the UH10 hiPSCs was approved by the Institutional Review Board at Guangzhou Institutes of Biomedicine and Health. K562, Jurkat and SUP-T1 cell lines were purchased from the American Type Culture Collection and were cultured in RPMI-1640 media with 10% fetal bovine serum (FBS, no. FSD500, ExCell Bio). Adult human peripheral blood was obtained from two patients with T cell acute lymphocytic leukemia (T-ALL) enrolled at the Department of Hematology, The Third Affiliated Hospital, Sun Yat-sen University. PBMCs were isolated by using human whole-blood mononuclear cell separation solution (YUANYE) according to the manufacturer's instructions. PBMCs were cultured in RPMI-1640 with 10% FBS. We have complied with relevant ethical regulations and this study was approved by the institutional review board at Guangzhou Institutes of Biomedicine and Health.

### CD5 CAR design

The CAR gene constructs include the CD8 signal peptide, the CD5 single-chain variable fragment (scFv) sequence, flag, CD8αhinge, and CD8 transmembrane structural domains, as well as the CD3ζ intracellular structural domains, concomitantly linked with an GFP sequence via a P2A sequence. The expression of which was regulated by the UBC promoter. There is no costimulatory domain. The GFP tag is to determine if the CAR structure is expressed. The FLAG tag is between the extracellular binding domain and the transmembrane domain to determine whether the CAR structure is located on the cell membrane. The CD5-scFv cDNA sequence, derived from a humanized murine immunoglobulin targeting CD5, was codon-optimized and cloned into a vector containing the remaining CAR components, all synthesized by Tsingke Biotechnology. In addition, a mutant CAR lacking the intracellular signaling domain CD3ζ was constructed.

### Virus production and transduction

Luciferase-P2A-DsRed, CD5, CD5-mcherry open reading frame was cloned into pSIN-EF1α-PGK-Puro lentiviral vectors; CD5-targeting CAR and CAR∆ constructions were cloned into pSIN-UBC-PGK-Puro lentiviral vectors. Lentiviral particles were produced by transfecting 293T cells with the packaging plasmids pMD2G and psPAX2. Virus was harvested 36 and 60 h after transfection and concentrated by ultracentrifugation at 50,000 relative centrifugal force for 2.5 h at 4 °C. Viruses were reconstituted with DMEM/F12 medium. Virus construct containing luciferase-P2A-DsRed open reading frame was transduced into cell lines K562, Jurkat and SUP-T1; CD5 were transduced into K562 and Jurkat; CD5-mcherry were transduced into Jurkat; CD5-targeting CAR and CAR∆ were transduced into hiPSCs. Transduction was carried out in 6-well plates when the confluent of the cells was ∼40%. After 48 h of infection, we selected target cells by FACS system for 100% DsRed positivity before use.

### Macrophage differentiation

hPSCs were dissociated by Accutase (Sigma) and plated on growth factor-reduced Matrigel (Corning)-coated plates with thiazovivin (0.1 μM, Selleck). hPSCs were induced for stepwise blood differentiation in basal medium supplemented with cytokines and inhibitors. Briefly, hPSCs were treated with DMEM/F12 (Thermo) medium containing 40 ng/mL BMP4 (Peprotech), 30 ng/mL ACTIVIN A (Peprotech), 20 ng/mL basic fibroblast growth factor (bFGF) (Sino Biological) for 1-2 days. Then, we switched to medium containing 40 ng/mL vascular endothelial growth factor (Sino Biological) and 50 ng/mL bFGF for 2 or 3 days. HSPCs were induced by medium containing 10 ng/mL SCF (Peprotech), 50 ng/mL thrombopoietin (Sino Biological), 10 ng/mL IL-3 (Sino Biological), 50 ng/mL IL-6 (Sino Biological), and 50 ng/mL FMS-like tyrosine kinase 3 ligand (FLT3L) (Peprotech). Floating iHSPCs were collected and plated in myeloid differentiation medium (StemPro +50 ng/mL FLT3L + 50 ng/mL M-CSF + 25 ng/mL GM-CSF) for 12 days and switched to macrophage maturation medium (RPMI-1640 + 10% FBS + 50 ng/mL M-CSF) for 7 days. All of the recombinant human cytokines were purchased from Sino Biological.

### Animal experiments

Animal experiment schemas are shown in detail in relevant figure. For scientific reasonableness and experimental repeatability, no less than 5 mice were assigned to each group. NCG mice were purchased from GemPharmatech and bred in the specific pathogen-free-grade animal care facility of the Guangzhou Institutes of Biomedicine and Health (GIBH), CAS. Bioluminescent imaging was performed using an IVIS Spectrum (PerkinElmer), and analysis was performed using LivingImage (Caliper Life Sciences). Mice were weighed and were subject to routine veterinary assessment for signs of overt illness. Animals were housed in SPF facilities with controlled temperature 22 °C, humidity 50%, and a 12-hour light/dark cycle.

### RNA extraction and quantitative real-time PCR

Total RNA was extracted with Trizol (Invitrogen), and complementary DNA was generated using oligo dT (Takara) and RT ACE (Toyobo). mRNA levels were quantified by real-time PCR using SYBR Green (Vazyme) and CFX96 machine (BIO-RAD) and were represented relative to GAPDH expression. All the data were measured in three duplicates.

### Western blotting with CD3ζ antibodies

The expression of the fusion protein CAR was detected using anti-CD3ζ antibodies. Cells were lysed using RIPA buffer RIPA buffer (Beyotime) loaded on 4-20% SDS-PAGE gel and transferred to PVDF membranes (Millipore) and incubated with with an antiCD3ζ mAb (1:1000) at room temperature for 2 h. The membranes were washed in TBST, incubated with HRP-labeled goat anti-rabbit IgG secondary Ab (1:2000). HRP was detected by ECL (Trans), and visualized by GelView 6000Plus (BLT).

### Flow cytometry

Cells were digested into single cells and suspended in PBS with 2% FBS. The cell suspension was incubated with the antibody for 30 min at 4 °C. After the incubation, the expression of the markers in this study were analyzed by the CytoFLEX Flow Cytometer (Beckman); for cell sorting, cells were sorted by the Moflo (Beckman), and then analyzed using FlowJo (Treestar). The following antibodies were purchased from BD Biosciences: CD11b-APC-Cy7, CD14-PE, CD80-APC, CD86-APC, CD163-PE, CD206-BV421 and CD5-APC.

### Phagocytosis assay

Cells were cultured in 96-well plates at a concentration of 10^5^ cells/mL in RPMI-1640 medium supplemented with 10% FBS and 1% penicillin-streptomycin. Carboxylate-modified red fluorescent latex beads with a mean diameter of 1 mm (L3030; Sigma-Aldrich) were added at a concentration of 1:200, and the cells were incubated with or without beads for 2 h. After repeated washing, the cells were analyzed by flow cytometry.

### Macrophage polarization

For M1 polarization, iMacs were exposed to 20 ng/mL recombinant IFN-γ (Sino Biological) and 100 ng/mL LPS (InvivoGen) in RPMI-1640 with 10% FBS for 24 h. As for M2 polarization, macrophages were exposed to 20 ng/mL recombinant human IL-4 (Sino Biological).

### Cytotoxicity Assay Based on Flow Cytometry

WT-iMac or iCAR-Ms cells were seeded into 24-well plates at a density of 9×10^4^ cells per well. Simultaneously, K562 or K562-CD5OE cells expressing DsRed fluorescent protein were added at an effector-to-target (E: T) ratio of 3:1. The cells were then cultured in an incubator at 37 ºC with 5% CO2. Every 24 hours, Cells from three replicate wells per group were harvested daily up to Day 5, washed with FACS buffer, and resuspended in 100 μl buffer. A fixed volume of 50μl of the cell suspension was aspirated for flow cytometry analysis. Cell populations were enumerated based on the flow cytometry results. Results were averaged across three replicate wells.

### *In vitro* cytotoxicity assay

Jurkat, Jurkat-CD5OE and SUP-T1 tumor cell expression Luciferase and DsRed were used as targets in the luciferase-based killing assays. WT-iMac and iCAR-Ms were used as effector. The effector-to-target (E:T) ratio was serially titrated from 10:1 down to 1:1. Bioluminescence was measured using an IVIS Spectrum (PerkinElmer). The luciferase signal (total flux) of the tumor-alone group was the control.

### Immunofluorescence analysis

Phagocytosis was defined as the complete internalization of an intact mCherry+ tumor cell within the cytoplasm of iCAR-Ms cell; Trogocytosis was identified by the presence of discontinuous, punctate, or fragmentary mCherry fluorescence signals—distinct from an intact cellular morphology—localized to the periphery or pseudopods of iCAR-Ms cells.

The cells were fixed with 4% paraformaldehyde. Images were captured with an LSM 800 microscope (Zeiss).

### Microscopy-Based Phagocytosis Assay

iCAR-Ms expressing green fluorescent protein (GFP) were plated at 10 × 10^4^ cells per well in 12-well plates. After 12 hours, K562-CD5OE cells expressing red fluorescent protein (mcherry) were added at E:T ratios of 5:1 or 1:2, followed by co-culture in a 37 °C incubator. Fluorescence imaging of mcherry and GFP was performed every hour using a fluorescence microscope (ZEISS, AXIO Vert.A1). For each group, three wells were used, and three random fields of view per well were selected to calculate the average number of phagocytosis or trogocytosis per field. All the data were measured in 9 duplicates.

### Live-Cell Imaging Video

iCAR-Ms expressing GFP were plated at 10 × 10^4^ cells per well on coverslips (NEST). After 12 hours, K562-CD5OE cells expressing mcherry were added at an E:T ratio of 1:1 and cultured in a humidity-controlled environment at 37 °C and 5% CO2 during acquisition. Fixed red and green fluorescence confocal plane images were collected every 30-60 seconds along the timeline using the SP8-STED microscope (Leica).

### RNA sequencing (RNA-seq)

iMacs, iCAR-Ms, mutant iCAR-Ms were lysed with TRIzol (Sigma) to isolated total RNA, and sequencing libraries were prepared by the TruSeq RNA Sample Prep Kit (Illumina) under the manufacturer's recommendations. The samples were run on a NextSeq system with the NextSeq 500 Mid Output kit (Illumina). RNA-seq data were processed as follows. Reads were aligned to an index generated from the Ensembl transcriptome version 74 (hg38) using HISAT2, and gene expression was analyzed with SAMtools and htseq-count, and normalized with EDASeq. A threshold of at least 20 average row read counts was used to filter lowly expressed transcripts. Differential expression was performed using DESeq2, and genes were considered significant if they had a Benjamini-Hochberg corrected p value < 0.05. Gene Ontology (GO) was performed using clusterProfiler, heatmap was prepared using pheatmap. The replicates of iCAR-Ms, iCAR∆-Ms was 3; the replicates of iMacs was 2.

### Statistical analysis

Statistical analysis was performed in GraphPad Prism 9. All of the central tendencies indicate the mean, and all of the error bars indicate SEM unless otherwise indicated. ANOVA multiple-comparison p values were generated using Tukey's multiple-comparisons test. All of the t tests were two-sided unless otherwise indicated. For all figures, ∗p < 0.05, ∗∗p < 0.01, ∗∗∗p < 0.001, and ∗∗∗∗p < 0.0001.

### Ethics declarations

All of the animal experiments were approved by the Institutional Animal Care and Use Committee at the Guangzhou Institutes of Biomedicine and Health. The ethical approval number is N2022087.

### Resource availability

All data are available in the main text or the supplementary materials. The RNA-seq data have been deposited in the GEO Database: GSE305641.

## Supplementary Material

Supplementary figures and tables.

Supplementary movie 1.

Supplementary movie 2.

Supplementary movie 3.
