# Supplementary material for: Identification of Trogocytosis as an Essential Limitation Factor in hPSC-derived CAR Macrophages
Source: Int J Biol Sci. 2026 Feb 26;22(6):3191–203. doi: 10.7150/ijbs.127434 (PMC13050468; doi:10.7150/ijbs.127434)
Supplement: Supplementary file 1 — Supplementary figures and tables. [file ijbsv22p3191s1.pdf]

Supplemental information

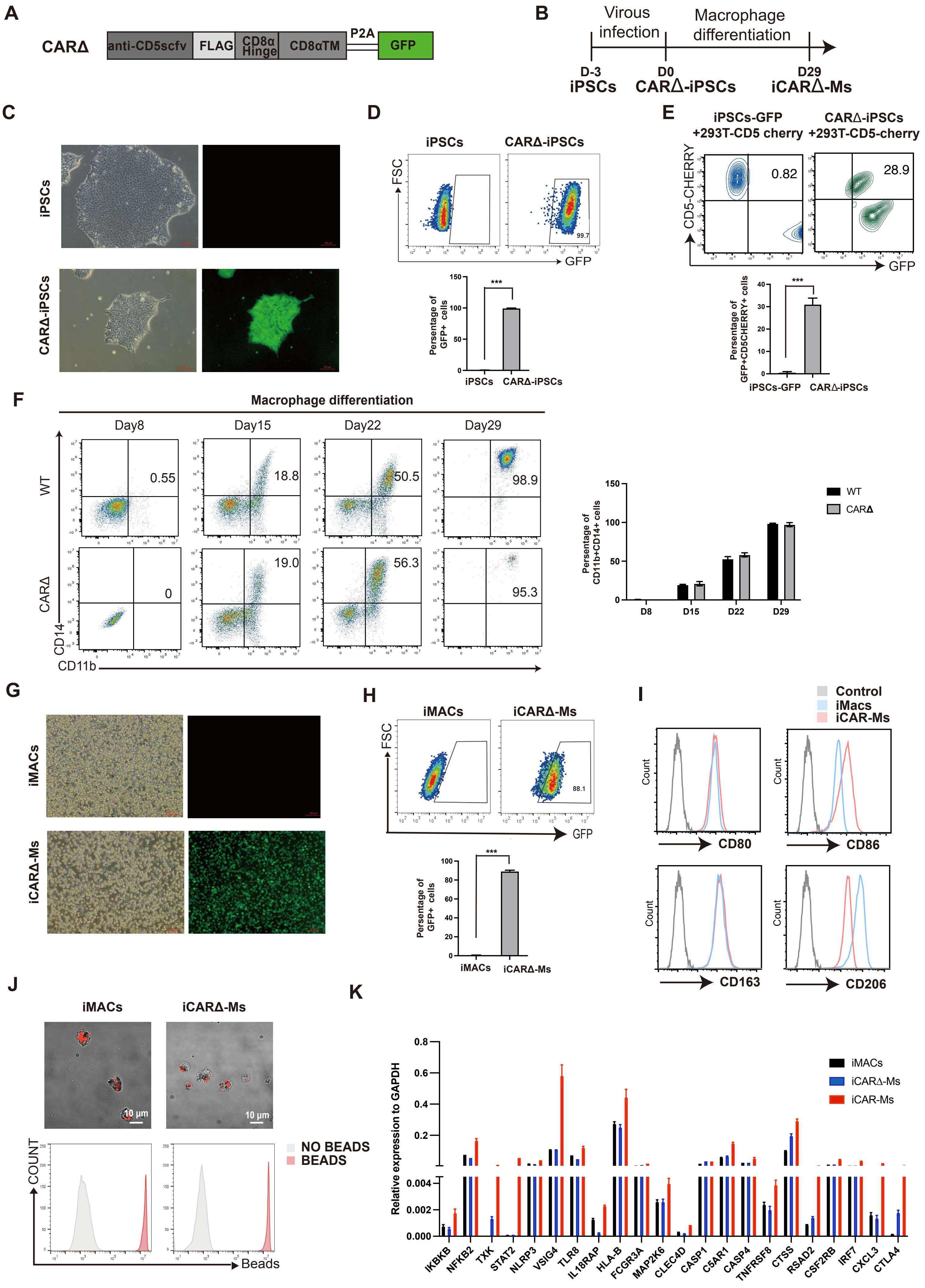

Figure S1. Generation of human iCARΔ-Ms targeting CD5. (A)Schematic representation of CARΔ constructs with CD5-targeting. (B) Schematic strategy for generating anti-CD5 CARΔ-Macrophage from human iPSCs. (C) The morphology and green fluorescent protein expression on indicated cells, Scale bar: 100μm. (D) FACS analysis of the iPSCs and GFP^{+}CARΔ-iPSCs. Statistics was determined using unpaired two-tailed Student's t-tests, ***$p < 0.001$. These data represent mean ± SD from three independent replicates ($n = 3$). (E) FACS analysis of the GFP^{+}CARΔ-iPSCs and $CD5cherry^{+}$293T-CD5cherry in coculture for 24h. Statistics was determined using unpaired two-tailed Student's t-tests, ***$p < 0.001$. These data represent mean ± SD from three independent replicates ($n = 3$). (F) FACS analysis of the indicated markers during macrophage differentiation. These data represent mean ± SD from three independent replicates (n=3). (G) The morphology and green fluorescent protein expression of indicated cells, Scale bar: 100μm. (H) FACS analysis the indicated macrophage markers in mature iMACs and iCARΔ-Ms. Undifferentiated human iPSCs serve as control. Statistics was determined using unpaired two-tailed Student's t-tests, ***$p < 0.001$. These data represent mean ± SD from three independent replicates ($n = 3$). (J) Engulf of red fluorescent latex labeled beads by iMACs and iCARΔ-Ms phagocytosis examined by confocal microscopy (left) and FACS. Scale bar: 10μm. **(K)** RT-qPCR analysis of the indicated gene expression of the iMACs, iCARΔ-Ms and iCAR-Ms cells. These data represent mean ± SD from three independent replicates ($n = 3$).

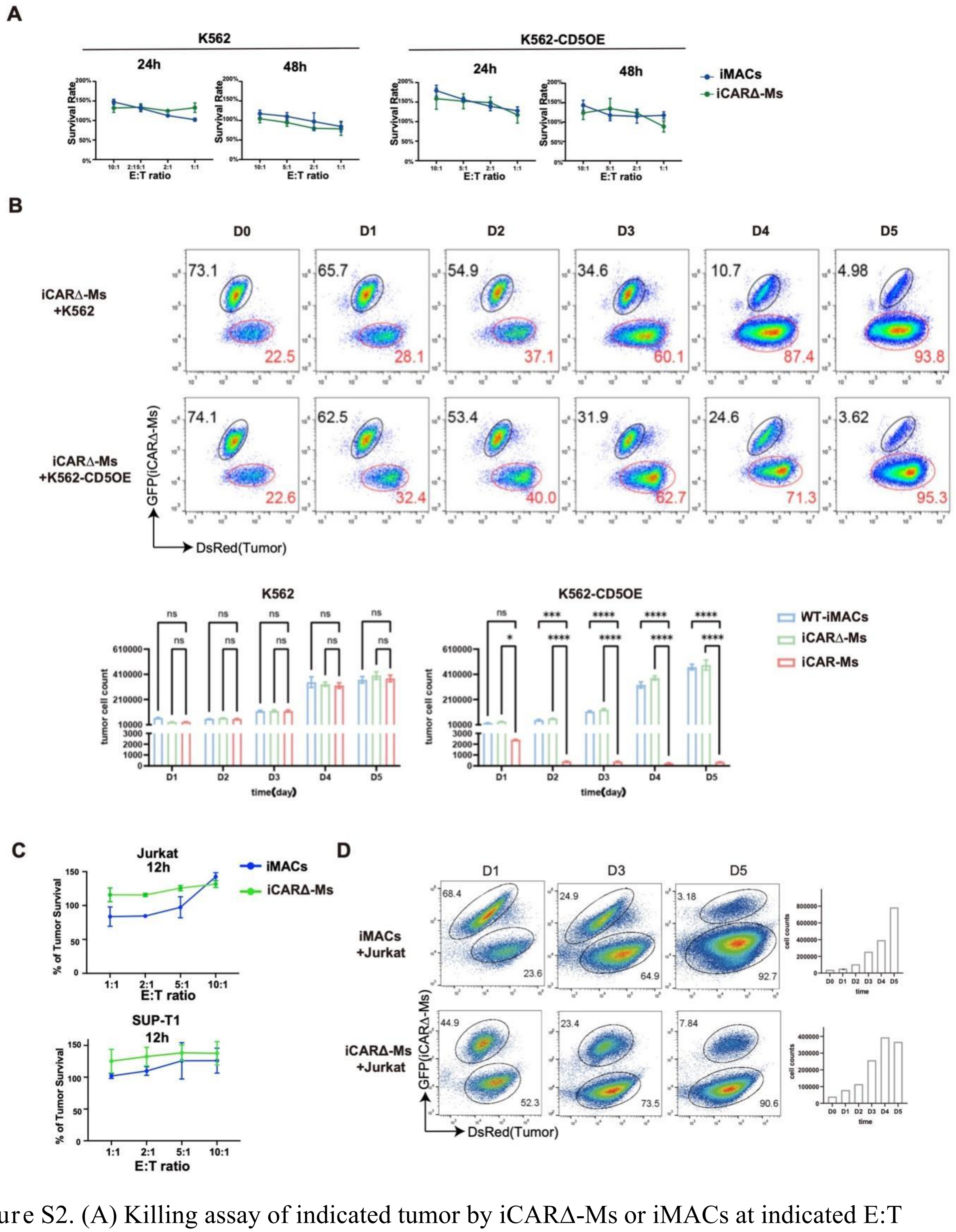

Figure S2. (A) Killing assay of indicated tumor by iCARΔ-Ms or iMACs at indicated E:T ratios. These quantitative data represent mean ± SD from three independent replicates (n=3). (B) FACS analysis of DsRed+ WT K562 or K562-CD5OE cells with iCARΔ-Ms(top). The remaining tumor cells were counted at indicated time point during co-culture(bottom). Statistics was determined using unpaired two-tailed Student's t-tests. These data represent mean ± SD from three independent replicates (n = 3). (C) Killing assay of indicated tumor by iCARΔ-Ms or iMACs after 12h of co-culture at indicated E:T ratios (bottom). These quantitative data represent mean ± SD from three independent

replicates (n=3). **(D)** FACS analysis of DsRed$^+$ WT K562 or K562-CD5OE cells with iCARΔ-Ms. The remaining tumor cells were counted at indicated time point during co-culture.

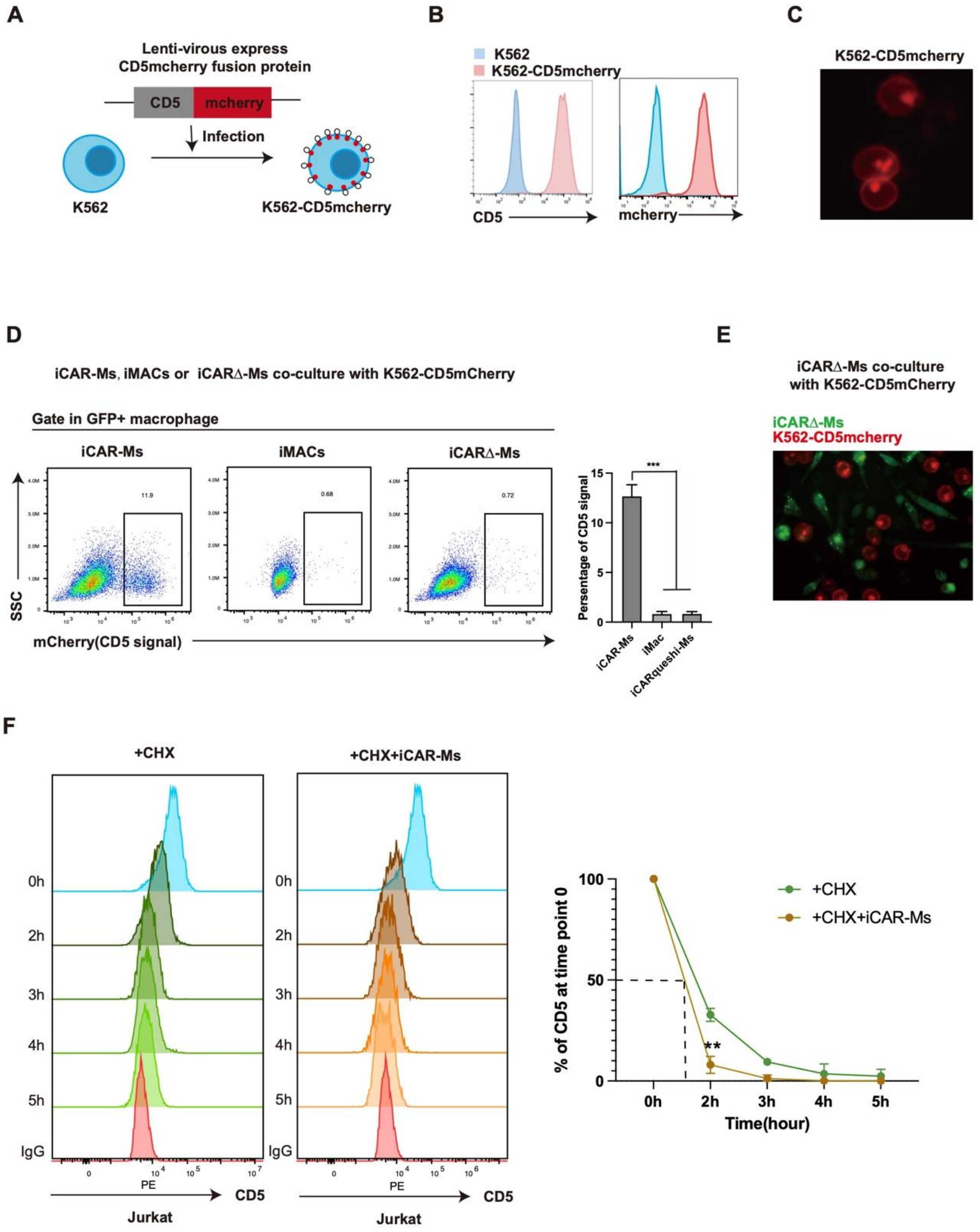

Figure S3. (A) Generation of K562 cells expressing CD5-mcherry fusion protein. Schematic representation of experimental design. **(B)** FACS analysis of CD5 and mcherry expression on K562-CD5mcherry, WT K562 as control. **(C)** Confocal analysis of mcherry expression on K562-CD5mcherry, Red, mcherry. **(D)** FACS analysis of mCherry^{+} CD5 of iCAR-Ms, iMACs and iCARΔ-Ms after co-culture with K562-CD5mCherry. **(E)**

Fluorescence microscopy images of iCARΔ-Ms and K562-CD5mcherry after co-culture 3h. Green: iCARΔ-Ms, Red: K562-CD5mcherry. Scale bar: 20μm. **(F)** Representative FACS analysis of CD5 and quantification of CD5 signal in Jurkat cells treated with 100 mg/mL cycloheximide (CHX) in the presence or absence of iCAR-Ms for the indicated times.

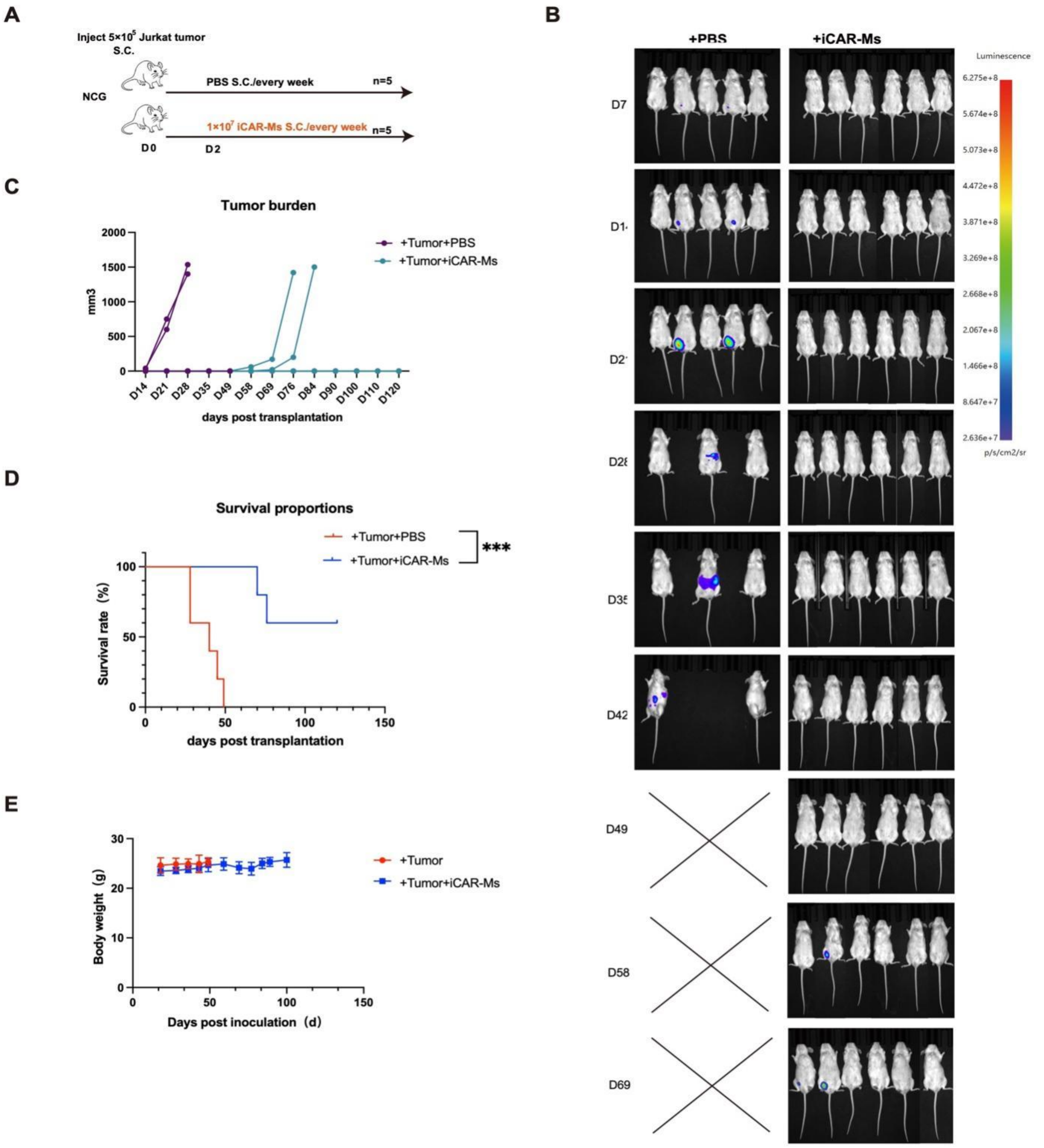

Figure S4.

(A) Experimental design: NCG mice were s.c. injected with luciferase_SUP-T1 and treated with s.c. PBS or iCAR-Ms as shown. **(B)** Imaging of luciferase-expressing SUP-T1 tumor-bearing mice. Tumor burden was measured by bioluminescence (total flux) at

indicated time. **(C)**Tumor burden monitored by measuring tumor volumes over 120 days. Each line represents 1 mouse. **(D)** Kaplan–Meier survival curve for animals injected with Jurkat tumor cells and indicated treatments. Statistical significance was calculated using the log-rank Mantel–Cox test. **(E)** Mouse weight monitoring. Data are represented as mean ± SD.

Movie S1. iCAR-M mediate trogocytosis on target tumor cells. iCAR-Ms stripped and internalized the cell-surface CD5-mCherry protein in co-culture. Green, iCAR-Ms; Red, K562-CD5mcherry. Scale bar: 10μm

Movie S2. iCAR-M mediate phagocytosis on target tumor cells. Green, iCAR-Ms; Red, K562-CD5mcherry. Scale bar: 10μm

Movie S2. iMACs has no interaction with tumor cells. iMACs(GFP overexpressing) co-culture with K562-CD5mcherry. Green, iMACs; Red, K562-CD5mcherry. Scale bar: 10μm

Supplemental Table 1 List of Chemicals and Antibodies

REAGENT	SOURCE	IDENTIFIER
Antibodies		
anti-CD3ζ antibody	Abcam	Cat# ab243874
Goat anti rabbit IgG HRP	Abcam	Cat# ab6721
HRP-conjugated GAPDH Monoclonal antibody	Proteintech	Cat# HRP-60004
Mouse Anti-Human CD5 APC	Elabscience	Cat# E-AB-F1041E
Mouse Anti-Human CD5 PE	BD Biosciences	Cat# 561897
Mouse Anti-Human CD11b APC-Cy7	BD Biosciences	Cat# 560914
Mouse Anti-Human CD14 PE	BD Biosciences	Cat# 561707
Mouse Anti-Human CD80 APC	BD Biosciences	Cat# 565157
Mouse Anti-HumanCD163 PE	BD Biosciences	Cat# 560933
Mouse Anti-HumanCD206 BV421	BD Biosciences	Cat# 566281
Mouse Anti-HumanCD86 APC	BD Biosciences	Cat# 560956
Chemicals and Recombinant Proteins		
DMEM/F12	Thermo Fisher Scientific	Cat# 11330-032
mTeSR1	Stem Cell Technologies	Cat# 85850
StemPro34	Thermo Fisher Scientific	Cat# 10639011
Matrigel	Corning	Cat# 354230
Penicillin/Streptomycin	Hyclone	Cat# SV30010
Y-27632	Selleck	Cat# S1049
TRIzol	MRC	Cat# TR118-500
DPBS	Thermo Fisher Scientific	Cat# 14190-144
FBS	ExCell Bio	Cat# FSD500
EDTA	Thermo Fisher Scientific	Cat# AM9262
Accutase	Sigma	Cat# A6964
RPMI-1640	Thermo Fisher Scientific	Cat# C11875500BT
thiazovivin	Selleck	Cat# S1459
human BMP4	Peprotech	Cat# 120-05ET
human ACTIVIN A	Peprotech	Cat# 120-14P-250
human bFGF	Sino Biological	Cat# 10014-HNAE
human VEGF	Sino Biological	Cat# 10008-HNAB-100
human SCF	Peprotech	Cat# 300-07-100
human thrombopoietin	Sino Biological	Cat# 13194-H08B-100
human IL-3	Sino Biological	Cat# 11858-HNAE-100
human IL-6	Sino Biological	Cat# 10395-HNAE-100
human FLT3L	Peprotech	Cat# 300-19-100
human M-CSF	Sino Biological	Cat# 11792-HNAH-20
human GM-CSF	Sino Biological	Cat# 10015-HNAH-20
LPS	InvivoGen	Cat# tlrl-peklps
human IFN-γ	Sino Biological	Cat# 11725-HNAS-20
human IL-4	Sino Biological	Cat# 11846-HNAE-5

Supplemental Table 2. Sequence of primers used for quantitative PCR

Gene	Forward primer	Reverse primer
IKBKB	GGAAGTACCTGAACCAGTTTGAG	GCAGGACGATGTTTTCTGGCT
NFKB2	ATGGAGAGTTGCTACAACCCA	CTGTTCCACGATCACCAGGTA
TXK	CATCCAGTCGGTTTTCTGTTGC	TGCGACGCTGGGTGTATTTT
STAT2	CCAGCTTTACTCGCACAGC	AGCCTTGGAATCATCACTCCC
NLRP3	GATCTTCGCTGCGATCAACAG	CGTGCATTATCTGAACCCCAC
VSIG4	GGGGCACCTAACAGTGGAC	GTCTGAGCCACGTTGTACCAG
TLR8	ATGTTCCTTCAGTCGTCAATGC	TTGCTGCACTCTGCAATAACT
IL18RAP	ATGCTCTGTTTGGGCTGGATA	GTGAGAGTCGATTTCTGTGGC
HLA-B	CAGTTCGTGAGGTTCGACAG	CAGCCGTACATGCTCTGGA
FCGR3A	CCTCCTGTCTAGTCGGTTTGG	TCGAGCACCCTGTACCATTGA
MAP2K6	GAAGCATTTGAACAACCTCAGAC	CCTGGCTATTTACTGTGGCTC
CLEC4D	CTGATACCTTCGGTTATTGCTGT	GCACTCCTGTGCCTCTCTTAC
CASP1	TTTCCGCAAGGTTCGATTTTCA	GGCATCTGCGCTCTACCATC
C5AR1	TCCTTCAATTATACCACCCCTGA	ACGCAGCGTGTTAGAAGTTTTAT
CASP4	CAAGAGAAGCAACGTATGGCA	AGGCAGATGGTCAAACTCTGTA
TNFRSF8	TCCACGGAGCACACCAATAAC	ACTGAGAGCATGACATCGCTG
CTSS	AAACGGCTGGTTTGTGTGC	CAGTGGTGATCCAGGGTAGG
RSAD2	TGGGTGCTTACACCTGCTG	GAAGTGATAGTTGACGCTGGTT
CSF2RB	AGCGGCTTCAGGACTCTTG	CTGGGCATGAGGTGCTCTG
IRF7	GCTGGACGTGACCATCATGTA	GGGCCGTATAGGAACGTGC
CXCL3	CGCCCAAACCGAAGTCATAG	GCTCCCCTTGTTCAGTATCTTTT
CTLA4	GCCCTGCACTCTCCTGTTTTT	GGTTGCCGCACAGACTTCA
